# The efficacy of topical prostaglandin analogs for hair loss: A systematic review and meta-analysis

**DOI:** 10.3389/fmed.2023.1130623

**Published:** 2023-03-14

**Authors:** Shangxuan Jiang, Zhuolun Hao, Wenli Qi, Zhenxing Wang, Muran Zhou, Nengqiang Guo

**Affiliations:** Department of Plastic Surgery, Union Hospital, Tongji Medical College, Huazhong University of Science and Technology, Wuhan, China

**Keywords:** hair loss, prostaglandin analog, Bimatoprost, Latanoprost, cetirizine, meta-analysis

## Abstract

**Background:**

Prostaglandin analogs have been found to have more versatile uses: treatment of open-angle glaucoma, high intraocular pressure, vitiligo, and other treatments. And prostaglandin analogs have been found to have an important role in the hair growth cycle. However, prostaglandin analogs have not been sufficiently studied for hair (including hair, eyelashes, and eyebrows) regeneration. In this study, a systematic review and meta-analysis of topical prostaglandin analogs on hair loss was performed.

**Objective:**

The purpose of this meta-analysis is to determine the efficacy and safety of topical prostaglandin analogs for treating hair loss.

**Methods:**

We searched PubMed, Embase, and Cochrane Library databases comprehensively. Data were pooled using Review Manager 5.4.1, and subgroup analyses were performed if necessary.

**Results:**

There were six randomized controlled trials included in this meta-analysis. All studies compared prostaglandin analogs with placebo, and one trial consisted of two sets of data. The results showed that prostaglandin analogs could significantly improve the hair length and density (*p <* 0.001). As far as adverse events are concerned, there was no significant difference between the experimental group and the control group.

**Conclusion:**

In patients with hair loss, the topical prostaglandin analogs have better therapeutic efficacy and safety than placebo. However, the best dose and frequency of experimental treatment require further studies.

## Introduction

Hair loss is one of the most anxiety-provoking and emotionally distressing dermatologic conditions. Psychological distress can be caused by hair loss. In addition to hair loss, eyelashes and eyebrows can also be lost. There is a range of severity when it comes to hair loss, from a single patch to multiple patches or more than 50% of the scalp. Hair loss not only affects esthetics but also profoundly affects a person’s mental state, and people with hair loss often lack self-confidence. It is important to differentiate between non-cicatricial and cicatricial alopecia. Non-cicatricial alopecia occurs more frequently. It is common for non-cicatricial alopecia to result from androgenetic alopecia, trichotillomania, and telogen effluvium. There are several types of cicatricial hair loss, including frontal fibrosing alopecia, folliculitis decalvans, and discoid lupus erythematosus (1). Hair loss also includes loss of eyelashes and loss of eyebrows. Hypotrichosis of the eyelashes refers to an insufficient amount of eyelashes (2). Eyelash hypotrichosis can be caused by many factors, including hereditary, age-related, chemotherapy, and unknown factors (2). There are other factors that can cause thin or absent lashes, including physical trauma to the face, eye surgery, trichotillomania and alopecia areata (3–5). In addition endocrinopathies, primary dermatoses, autoimmune conditions, infections, neoplasms, genetic conditions, and exogenous agents can cause eyebrow hair loss (6).

To date, there are several non-surgical ways to regrow hair, but the results are variable. These include topical or oral finasteride, minoxidil, dexpanthenol treatment, PRP, microneedle therapy, laser therapy, etc. (7–9). Although the above treatments are in some ways better (10, 11) for hair loss, they still have many shortcomings. It has been shown that finasteride increases the risk of sexual dysfunction in multiple randomized trials involving placebo controls (12, 13). In the beginning of minoxidil treatment, paradoxical hair shedding may occur (14). Contact irritant dermatitis and facial hirsutism (15, 16) are also seen after topical application. PRP efficacy data cannot be compared between studies, which is a major limitation in interpreting them. The effectiveness of micro-needling as a monotherapy is uncertain, though it may have some benefit when combined with other hair growth stimulants (17, 18).

Due to the lack of proven therapies, we thought of prostaglandin analogs. Treatment with topical prostaglandin analogs has recently shown better efficacy than conventional therapies (19–27).

Prostaglandins produced by almost all tissues and organs in the body are local hormones, except red blood cells. They are active at the location of their secretion or near the cells that secrete them. It is thought that prostaglandins play an important biological role in both physiological and pathological processes (28, 29). Prostaglandin analogs have a similar chemical structure to prostaglandins and have similar biological activity. Prostaglandin analogs are possible treatments for eyelash alopecia. These drugs can also affect fibrosis alopecia and alopecia areata, such as eyebrow hypotrichosis. Hypertrichosis induced by prostaglandins appears to occur by inducing follicles into anagen phase. It is essential for hair growth to be regulated by prostaglandins (PGs) (30). The scalp of people with AGA has decreased levels of PGE2 and increased levels of PGD2. Hair growth is inhibited by PGD2, while it is stimulated by PGE2 and PGF2a (31). Clinically known prostaglandin analogs include Bimatoprost, Latanoprost, and Cetirizine (32, 33). Synthetic analogs of PGE2 are known as Bimatoprost. The topical application of bimatoprost 0.03% lotion daily for 12 and 16 weeks has been found to significantly increase the diameter of vellus hair and the number of vellus hairs (34).

Synthetic analogs of PGF2a are known as Latanoprost. It was found that latanoprost 0.1% applied daily during 24 weeks resulted in significant improvements in hair density compared with baseline in 16 male patients suffering from AGA (35).

Studies have shown that cetirizine decreases PGD2 production. The effectiveness of topical 1% cetirizine applied every day for 6 months was evaluated in pilot studies on 85 patients with hair loss (36). According to the results, cetirizine could significantly increase both terminal hair density and total hair density.

However, the number of patients in all these studies was small. Therefore, there is some evidence that these drugs are effective, but further research is needed. In order to answer this question, randomized controlled trials were conducted to evaluate the efficiency and security of topical prostaglandin analogs. The purpose of this study was to use a systematic review and meta-analysis to identify a more novel approach for the treatment of hair loss and to analyze the efficacy and safety of this method.

## Methods

Meta-analysis was carried out in compliance with the *Preferred Reporting Items for Systematic Reviews* and Meta-analyses (PRISMA) statement. And Our meta-analysis had been registered on the official PROSPERO website with the registration number *CRD42022365811*.

### Search strategy

**Figure. 1:**
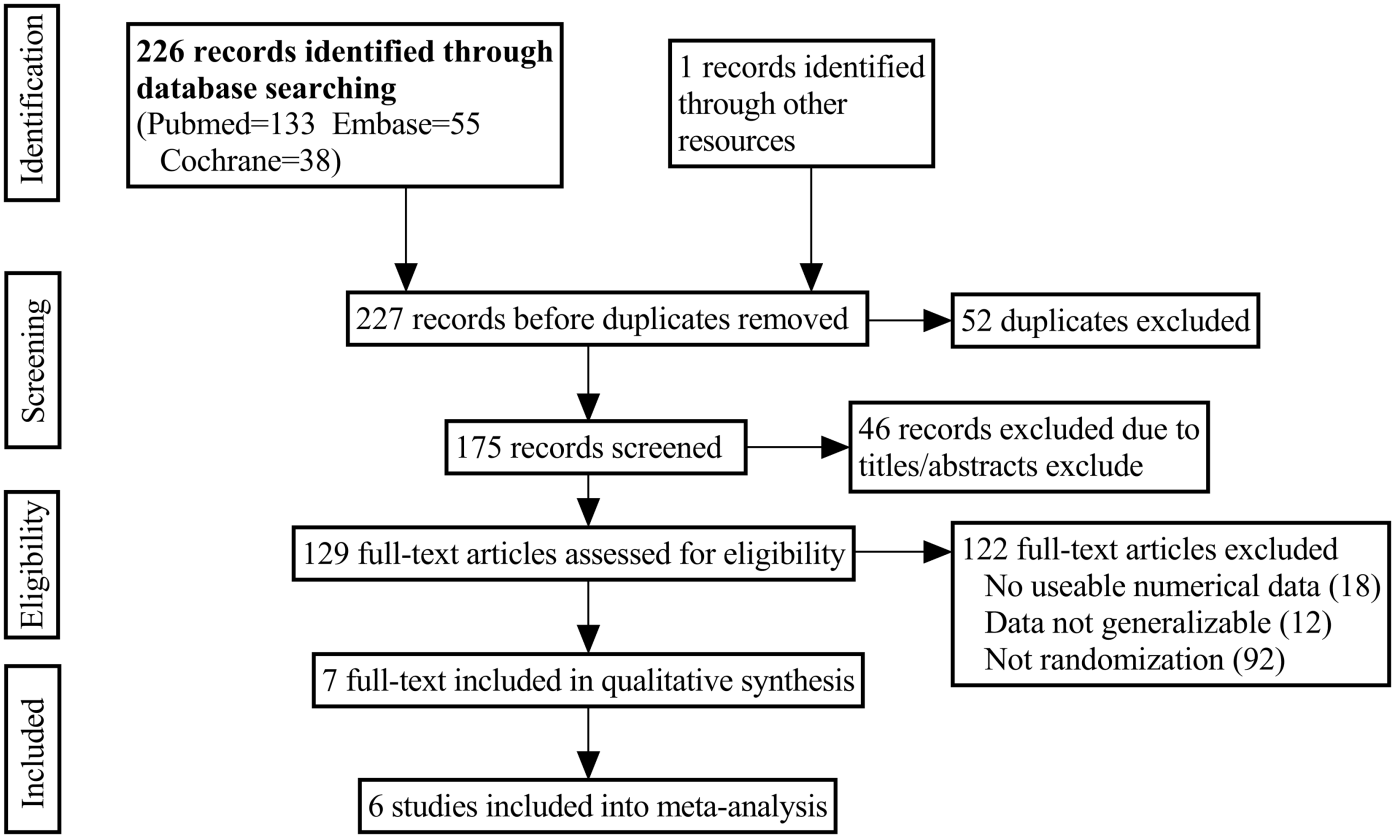
Selection procedure and search results.

A number of electronic databases were searched to find potentially relevant studies, including PubMed, Embase, and the Cochrane Library, from inception to September 2022. Combining these keywords with the Boolean operators “AND” or “OR” yielded the following results: “alopecia OR hair loss,” “prostaglandins,” “bimatoprost,” “cetirizine,” “latanoprost.” Language restrictions were not imposed. Additional publications were identified by cross-referencing other related publications and trials retrieved in the bibliographies. The search process was shown in Figure 1.

### Inclusion criteria

Those trials meeting the PICOS criteria (patients, intervention, comparator, outcome, study design) were included in this meta-analysis. (1) Patients: Men or women with hair thinning. (2) Intervention: using topical prostaglandin analog (Bimatoprost, Latanoprost, Cetirizine). (3) Comparator: Topical treatment with placebo alone. (4) outcomes: change in hair length or density (5) Study design: randomized controlled trial(RCT). A retrospective study, a letter, a guideline, a surgical registry, a review paper, or a study with insufficient outcome data were excluded from the analysis.

### Data extraction

Two reviewers independently extracted related information from the articles with a standard data extraction table. Data extracted included information about authors, study designs, sample sizes, publication dates, ages, and outcomes. The primary outcomes included the change in hair length or density, which was a comprehensive photographic assessment performed by the investigators at the end of treatment. In cases where the eligibility criteria were unclear, we contacted authors for additional information. Whenever there was disagreement between the two authors, a consensus was reached through discussion.

### Assessment of methodological quality

Based on the guidelines in the *Cochrane Handbook for Systematic Reviews of Interventions*, independent assessment of the quality of the included studies was conducted by the authors. The following contents were included in our “risk-of-bias” table: selection bias(random sequence generation and allocation concealment), blinding, reporting bias(selective reporting), attrition bias(incomplete outcome data), and other bias. There were three categories of bias risk evaluated in each study: “low risk of bias,” “unclear risk of bias,” or “high risk of bias.”

### Statistical analysis

To conduct the meta-analysis, we used Review Manager 5.4.1 software. Continuous outcomes were assessed with mean differences (MD) and 95% confidence intervals, and outcomes were assessed with odds ratios (OR). Meta-analysis of variables was performed when the outcome was reported by two or more studies. The heterogeneity of studies included in this study was assessed using *p* and *I*^2^, with a fixed-effects model when *I*^2^ < 50% and *p* > 0.1; otherwise, a random-effects model was used. Statistical significance was determined when the *p* value was less than 0.05.

## Results

### Search results

Figure 1 illustrated the PRISMA flow chart used for the study search. A total of 227 studies from the database were searched from the search strategy. A total of 98 articles were excluded after removing duplicates and screening by titles and abstracts. Eighteen studies were excluded because they lacked usable numerical data after a detailed reading of their full texts. Ninety two studies were excluded due to non-RCT. And 12 studies were excluded because the experimental data were not generalizable. Finally, six studies (35–40) met the inclusion criteria, including 683 patients.

As shown in Table 1, an overview of the basic characteristics and interventions was provided. Three RCTs (37–39) compared the efficacy of Bimatoprost with that of the control group, two RCTs (35, 40) compared the efficacy of Latanoprost with that of the control group, and one RCT (36) compared the efficacy of Cetirizine with that of the control group. In all relevant RCTs, topical Bimatoprost, Latanoprost, and Cetirizine were administered 1 or 2 times/day at doses of 0.05 mL, 0.25 mL, and 1 mL. Changes in hair length or density were calculated in 6 RCTs. Six RCTs had varying end-of-follow-up times, ranging from as short as 12 weeks to as long as 12 months.

**Table 1:** Characteristics of included studies.

Author	Year	Site	Age (Mean ± SD)	Number	Severity	Causes of hair loss	Duration	Frequence (daily)	Dose	Change in length or density		Mode of assessment	Side effects (*n*)
Gurpreet S. Ahluwalia et al.	2013	eyelash	NA	Bimatoprost:96 Placebo:34	severe	chemotherapy	12 m	1	0.05 mL	Placebo:35.6	digital image analysis	Bimatoprost:12 Placebo:0	Bimatoprost:39.1
A. Rossi et al.	2017	scalp	20–65	Cetirizine:67 Placebo:18	NA	androgenetic alopecia	6 m	NA	1 mL	Placebo:2.59 ± 0.55	global photographic	NA	Cetirizine:30.31 ± 4.21
Ulrike Blume-Peytavi et al.	2012	scalp	23 to 35	Latanoprost:16 Placebo:16	mild	androgenetic alopecia	24 w	1	0.05 mL	Placebo:0.10	global photographic	Latanoprost:6 Placebo:1	Latanoprost:0.22
Mohammadreza Rafati et al.	2022	scalp	Latanoprost:29.2 ± 9.8 Placebo:29.8 ± 12.1	Latanoprost:12 Placebo:12	NA	alopecia areata	12 w	1	0.25 mL	Placebo:14.6 ± 18.6	global photographic	the same between groups	Latanoprost:37.2 ± 26.1
Mark Borchert et al.	2016	eyelash	Bimatoprost:14.5 Placebo:14.6	Bimatoprost:48 Placebo:23	GEA < 3	multiple factors	4 m	1	0.05 mL	Placebo:0.79	digital image analysis	Bimatoprost:12 Placebo:0	Bimatoprost:1.21
Jean Carruthers et al.	2016	eyebrow	Bimatoprost BID:53.5 ± 10.82 Bimatoprost QD:55.0 ± 11.11 Placebo:53.5 ± 9.8	Bimatoprost BID:118 Bimatoprost QD:118 Placebo:121	GEBA≤2	NA	7 m	1 or 2	0.05 mL	Placebo:6.42	digital image analysis	Bimatoprost BID:42 Bimatoprost QD:48 Placebo:41	Bimatoprost:

GEA, Global Eyelash Assessment; GEBA, Global Eyebrow Assessment; QD, Bimatoprost once daily; BID, Bimatoprost twice daily.

### Risk-of-bias assessment

**Figure. 2:**
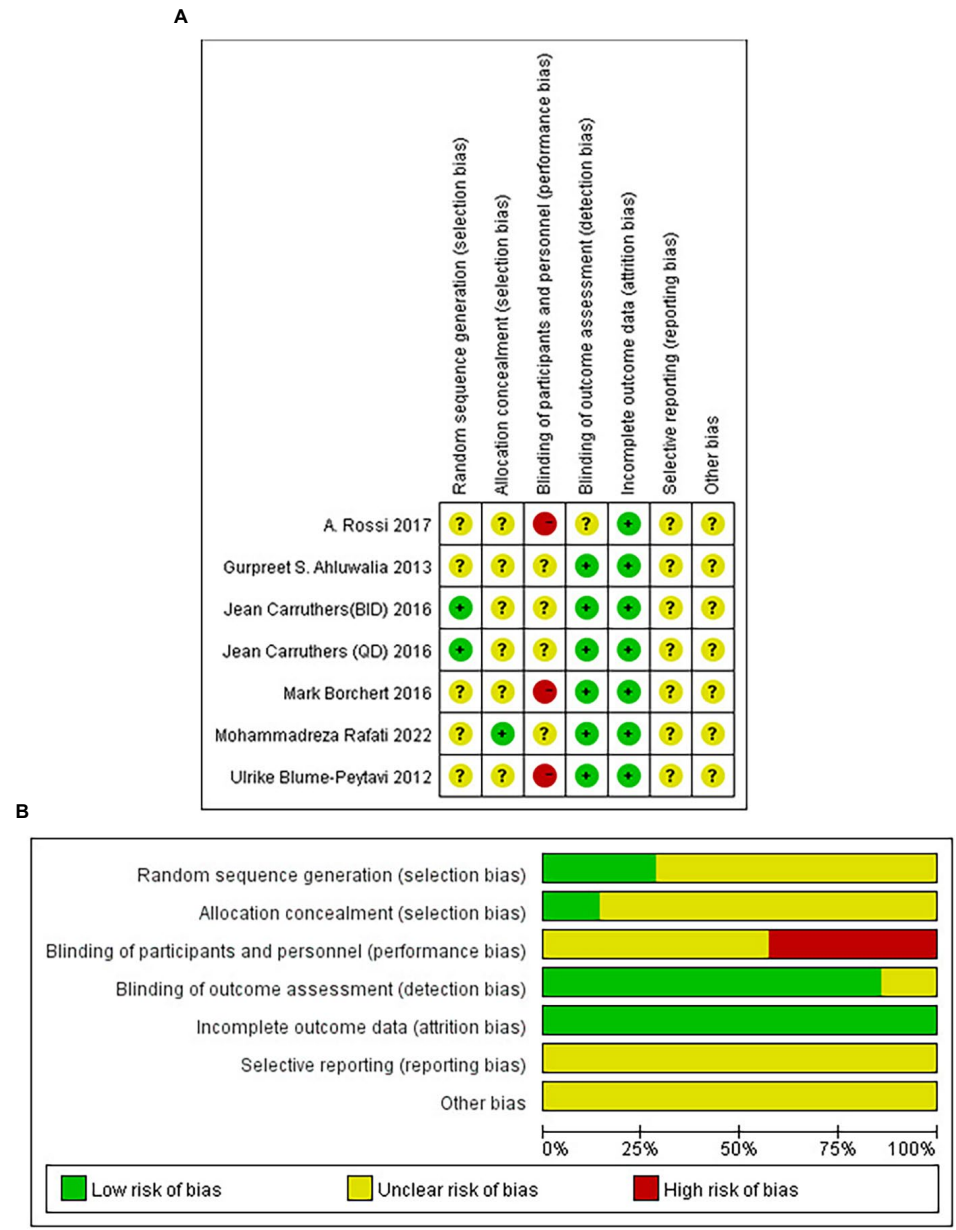
Risk-of-bias summary **(A)** and graph **(B)** of the included studies.

Figure 2 illustrated the risk of bias risk assessment. Among the six RCTs, only one (39) used the method of randomizing based on a random number table, and the other five (35–38, 40) did not describe how randomization was generated. Allocation concealment was not mentioned in any of these studies except for Mohammadreza Rafati’s experiment (40). Five studies (35, 37–40) described that the study results were assessed by two observers who were unaware of the trial method, and one study was not mentioned (36). A total of six studies submitted complete data, and none of the included studies showed selective reporting. A meta-analysis that included only 6 RCTs is difficult to assess in terms of publication bias since there were only 6 included.

### Results of the meta-analysis

#### Change in hair length or density

**Figure 3:**
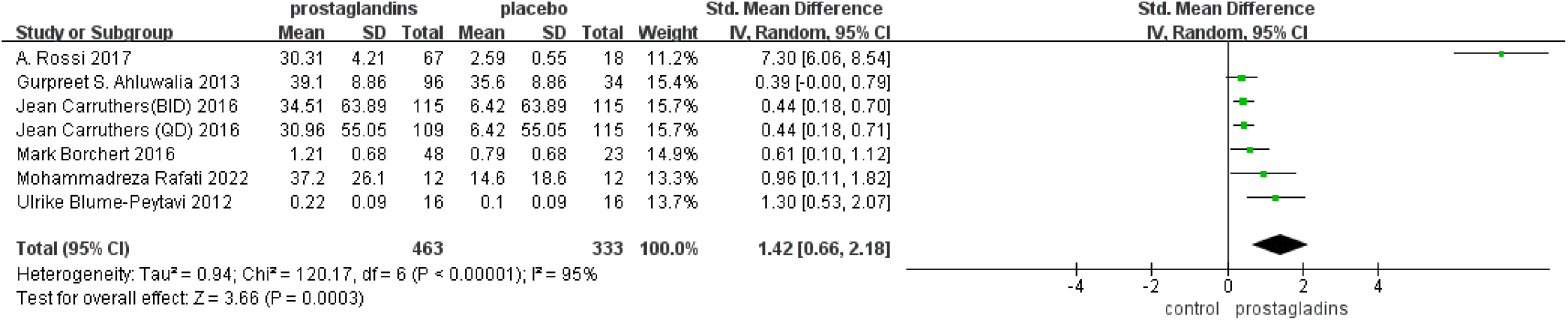
Forest plot diagram showing the Change in hair length or density at treatment end.

There were six studies that reported changes in hair length or density from baseline to the end of treatment (35–40), including 683 patients who compared placebo treatment. The experimental data from one paper were divided into two groups because of the difference in the frequency of dosing (39), so that we had a total of seven data sets for analysis. Based on a random-effects model, the meta-analysis found significant differences between the two groups[SMD =1.42, 95%CI (0.66, 2.18), *p* = 0.0003; Figure 3]. All test groups had more hair length or density growth than the control group. For example, Gurpreet S. Ahluwalia’s trial (37) showed a 39.1% increase, higher than the 35.6% increase in the control group. The subjects in A. Rossi’s test group (36) had a hair growth of 30.31 ± 4.21 mm compared to 2.59 ± 4.21 mm in the control group.

**Figure 4:**
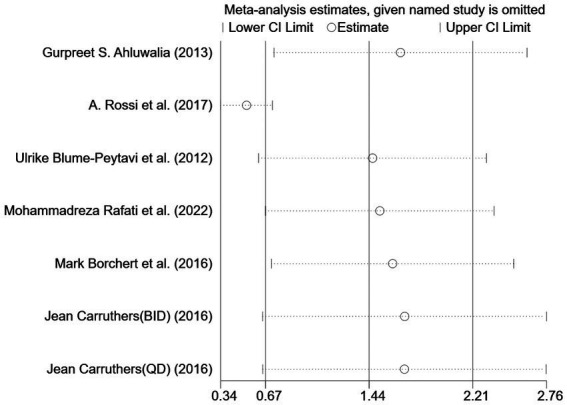
Sensitivity analysis.

However, the *I*^2^ statistic indicated excessive heterogeneity was existing among these six studies. We then performed a sensitivity analysis (Figure 4). We could find a significant deviation between the data of the article published by A Rossi et al. in 2017 and other data. After excluding this literature, the *I*^2^ value was 18%, which was less than 50%. After a closer look at A Rossi’s experiment, we speculated that it is the significant difference between the dose of their experiment and the other groups; they used a 1 mL experimental reagent, which is much larger than the other groups, which may have led to the bias of the data.

**Figure 5:**
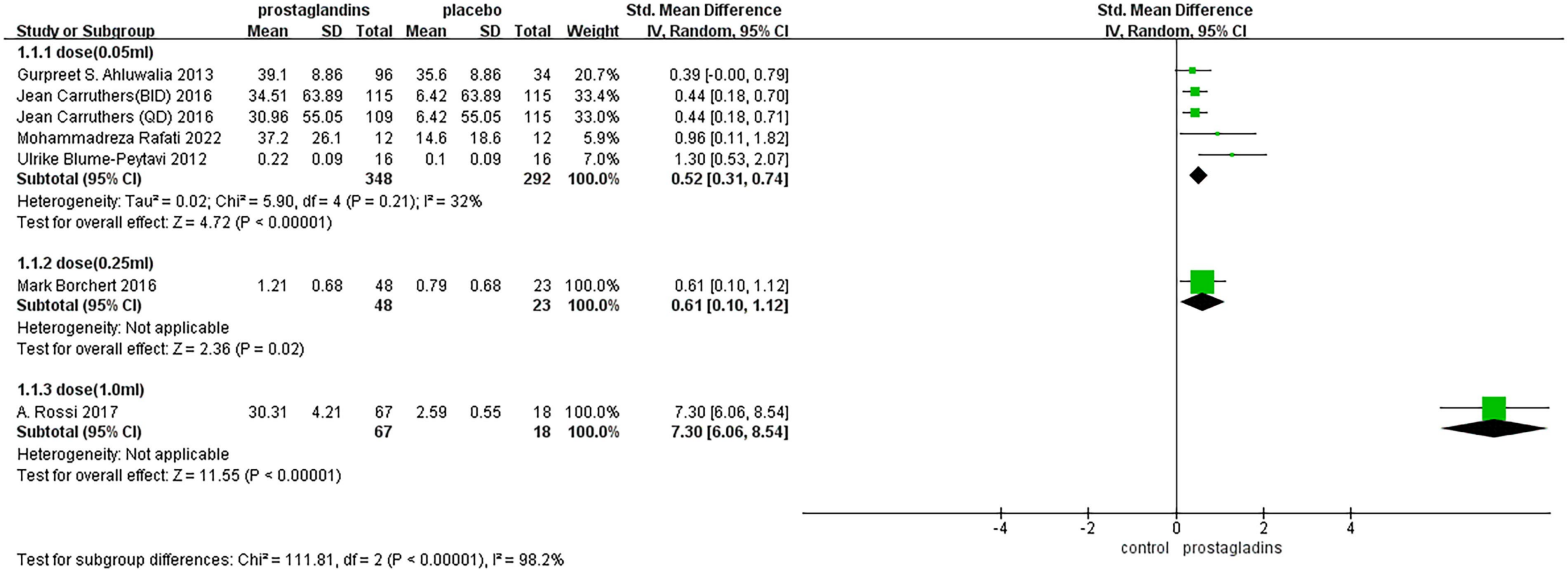
Forest plot diagram showing the subgroup at the treatment end.

The subgroup analysis of the dose was performed in each group (Figure 5), with 0.05 mL of experimental group reagent in all four experimental groups (35, 37, 39, 40), 0.25 mL of reagent in one experimental group (38), and 1.0 mL of reagent in one experimental group (36). The results showed that the dose of the 1.0 mL group had the largest effect size [SMD = 7.30, 95% CI (6.06, 8.54), *p* < 0.00001; Figure 5]. In contrast, in the dose of the 0.25 mL group, the effect size was not significantly different from that of the dose of the 0.05 mL group.

**Figure 6:**
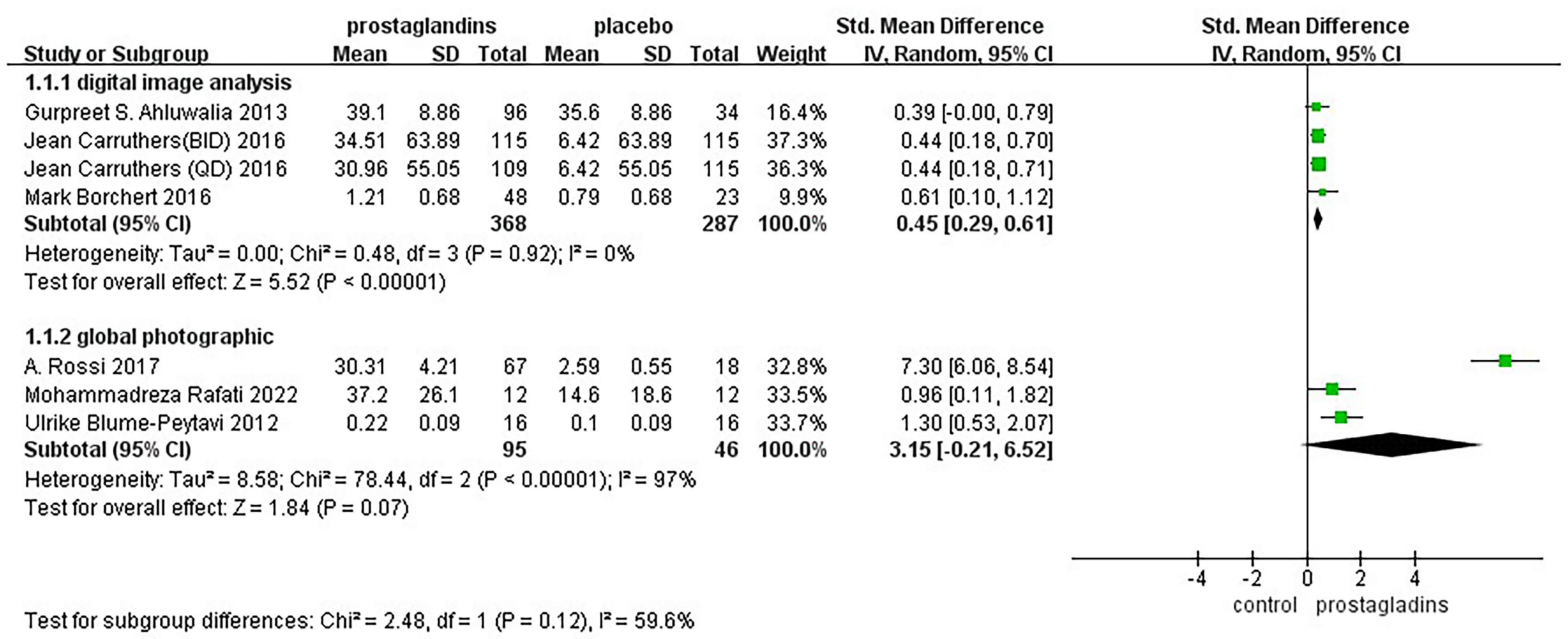
Forest plot diagram showing subgroup analysis of mode of assessment.

Considering that the way in which the efficacy was assessed may affect the results of the experiment, we performed a subgroup analysis of mode of assessment as well (Figure 6). It was divided into two groups, digital image analysis group and global photographic group. The results of the analysis showed low heterogeneity of results in digital image analysis group [SMD = 0.45, 95% CI (0.29, 0.61), *p* < 0.00001; Figure 6].

**Figure 7:**
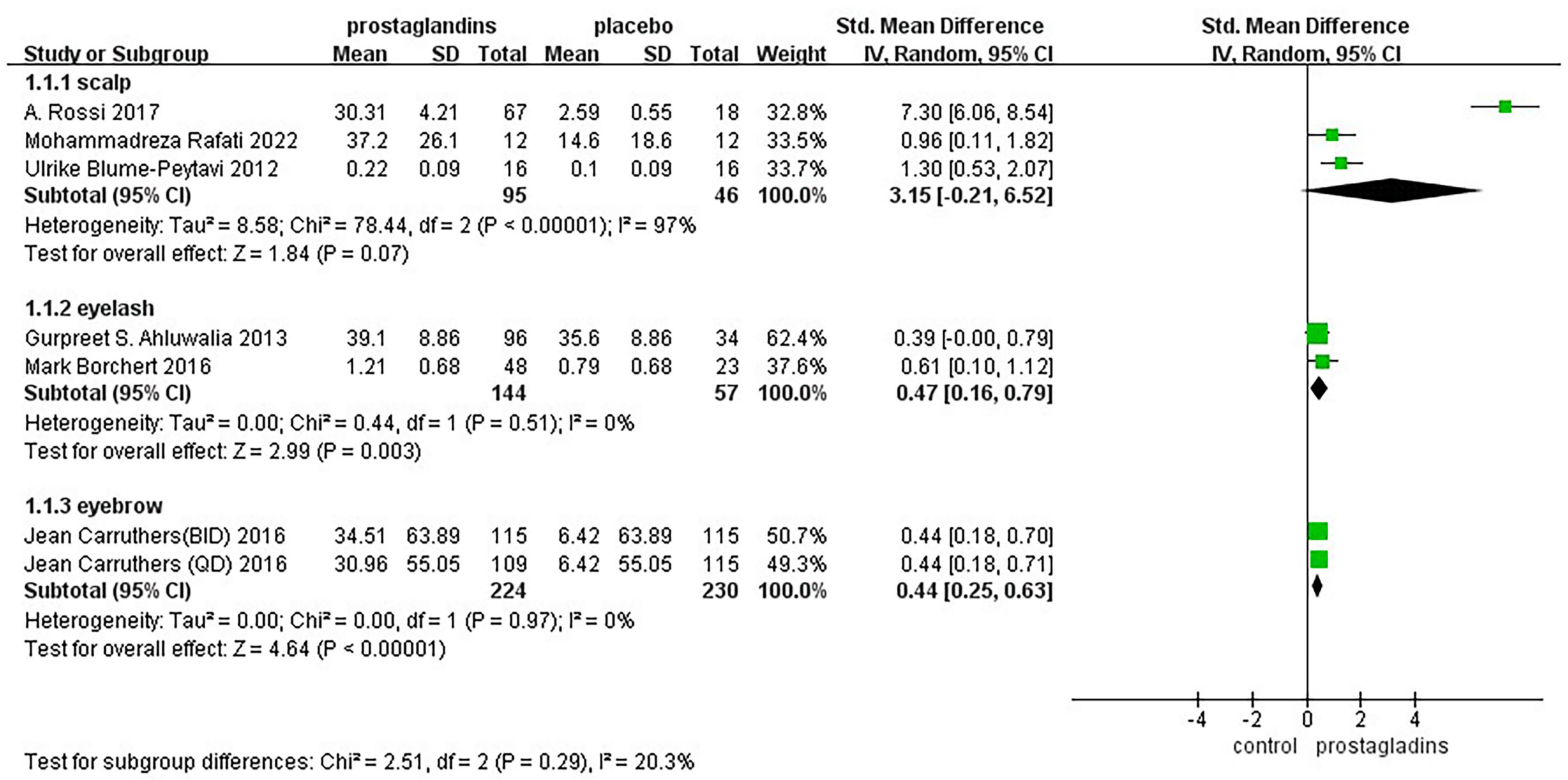
Forest plot diagram showing subgroup analysis of the site of hair loss.

To assess whether hair loss at different sites could be combined for analysis, we performed a subgroup analysis between groups for eyebrows, eyelashes, and scalp (Figure 7). The results showed fair heterogeneity between groups(*I*^2^ *= 20.3%*).

#### Adverse events

**Figure 8:**
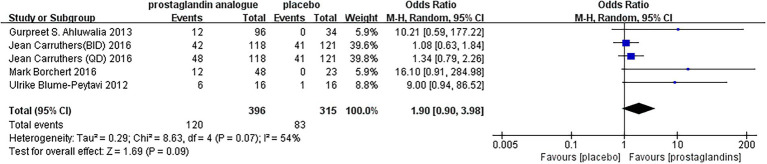
Forest plot diagram showing adverse effect.

During the follow-up period, adverse events were reported in five RCTs. As seen by the forest plot (Figure 8), MD = 1.90, 95% CI (0.90, 3.98), *p* = 0.07, *I*^2^ = 54%. It could be seen that there was still a slight increase in the incidence of adverse reactions after using prostaglandin analogs compared to using a placebo. But the gap and the severity of the adverse reactions were still within the acceptable range. Upper respiratory tract infections were reported in Two studies (37, 39), including 332 patients. There was 1 study with unknown adverse reactions (36). The remaining studies (35, 38, 40) reported local adverse reactions to drug administration, including conjunctival hyperemia, punctate keratitis, erythema, folliculitis, the sensation of bilateral burning, and application site pruritus. There were also rare side effects reported by enrolled studies, such as urinary tract infections, other body hair increase, influenza and actinic keratosis.

## Discussion

Psychological distress can be caused by hair loss, which is a common condition. Recently, the previous literature suggested that prostaglandin analogs, including Bimatoprost (34), Latanoprost (41), and Cetirizine (42), could promote hair growth in clinical trials. As far as we know, We have conducted the first systematic review and meta-analysis of prostaglandin analog therapy for hair loss on this topic. The aim of this meta-analysis was to assess the efficacy and safety of topical prostaglandin analog treatments in patients with a lack of hair(scalp, eyelash and eyebrow).

When searching the literature, we excluded case–control studies retrieved because historical information is often not verifiable and it is sometimes impossible to guarantee the authenticity of the information. Similarly, we excluded cohort studies because they are prone to missing visit bias, and changes in known variables or the introduction of unknown variables can also produce bias, thus reducing the credibility of the original data. Our meta-analysis involved 6 RCTs (35–40)(7 data sets). The data showed more significant improvement in hair regrowth in the topical prostaglandin analog treatment group, with more patients having a substantial increase in hair and a similar incidence of adverse events, suggesting that topical prostaglandin analog treatment is a better option for patients with a lack of hair (*p <* 0.001).

As shown by whole-body photographic evaluation, changes in hair length or density were a common way of evaluating efficacy. Prostaglandin analogs were found to promote superior hair growth in all studies compared to controls. Thus, our meta-analysis showed a significant advantage of topical prostaglandin analog treatment in hair regrowth (*p* < 0.001). It is possible that including more randomized controlled trials would not change this result. Using fixed-effects and random-effects models, we found utterly different results for hair density changes. This may be due to the fact that the number of randomized controlled trials is limited, and therefore, there was still a need for more studies to confirm these findings. In conclusion, our pooled data suggest that the topical prostaglandin analog treatment group has excellent results in hair regeneration and offers a new possible therapeutic prospect for patients with hair loss.

We likewise found that the effects of different doses of prostaglandin analogs on hair differed considerably. We performed a subgroup analysis of dose and could see that dose and effect size were positively correlated within the statistical range. However, we have less data to find the most appropriate dose.

The hair follicles are complex micro-organisms with constant cycling behavior associated with growth, quiescence, and involution (31). The growth and cycle of hair is influenced by numerous gene products and growth factors. The prostaglandins are unsaturated carboxylic acids composed of 20 carbon skeletons that are synthesized by biochemical means from arachidonic acid. According to Colombe (2007), human hair follicles are capable of metabolizing PGE2 and PGF2a (43). It was found in hair follicles a year later that all four major PG receptors are present and according to a 2012 study, PGD2 and its enzyme synthase were elevated in skin biopsies taken from male bald head scalps (44, 45). Then, the results of Eleni Chovarda’s study (46) indicated that a potential treatment for hair loss might target prostaglandin receptors, which possibly contributing to hair loss pathogenesis. We then analyzed the data from existing clinical studies and concluded that topical application of prostaglandin analogs may have a positive improvement in hair loss.

Generally, hair growth evaluation and follow-up are limited to individual physician and patient assessments. The investigators’ hair growth assessments were confirmed by global photographic assessment and Automatic digitalized photographic systems. In global photographic assessment of hair growth, experts are blinded to treatment and time and evaluate global photographs. With the help of a digital image analysis system, it is possible to quantify hair density, hair thickness, anagen hair ratio in the survey area (14). As with the results of the subgroup analysis described above, it was evident that the use of digital image technology allowed for a more accurate assessment of efficacy.

The cause of hair loss may influence treatment, and the causes of hair loss we included in our study were chemotherapy (37), alopecia areata (40), androgenic alopecia (35, 36), and a mixture of factors (38). The cause of hair loss in one of the included studies was unknown (39). For the included studies, prostaglandin analogs can treat the above causes of hair loss, but more data were needed to support this.

A few limitations are present in our review. First, we did not include all medical databases, such as Web of Science, in our search, and we included only English-language publications. As a result, this could lead to a selection bias. Second, the number of limited trials for each result prevented us from performing a funnel plot analysis. Third, the small number of our study and the small sample of two types of hairs, eyelashes and eyebrows, may affect the credibility of the results.

## Conclusion

Based on the six RCTs clinical trials with small sample sizes, the prostaglandin analogs showed better efficacy than placebo in hair loss treatment. However, the optimal dose of prostaglandin analogs treatment requires additional studies. In addition, the safety of topical application of prostaglandin analogs cannot be better guaranteed. Considering its efficacy and safety, hair loss disorder may be treated with topical prostaglandin analogs, particularly in patients who have not responded well to other forms of treatment. Further studies are also needed to better understand factors influencing prostaglandin analogs treatment outcomes in patients with hair loss. Among the concerns that might be taken into account are: sufficient sample size, optimal concentrations and regimens, and duration of topical prostaglandin analogs, adequate follow-up time, and standardized and appropriate outcomes, including patient satisfaction evaluations.

## Data availability statement

The original contributions presented in the study are included in the article/supplementary material, further inquiries can be directed to the corresponding authors.

## Author contributions

SJ: conceptualization, methodology, software, and writing–original draft. ZH: validation, data curation, and supervision. WQ: validation and resources. ZW: formal analysis and resources. MZ: writing–review and editing. NG: visualization and writing–review and editing. All authors contributed to the article and approved the submitted version.

## Funding

This study was funded by Natural Science Foundation of Hubei Province, China (No. 2019CFB561) and Youth Fund of National Natural Science Foundation of China (02.07.21020088).

## Conflict of interest

The authors declare that the research was conducted in the absence of any commercial or financial relationships that could be construed as a potential conflict of interest.

## Publisher’s note

All claims expressed in this article are solely those of the authors and do not necessarily represent those of their affiliated organizations, or those of the publisher, the editors and the reviewers. Any product that may be evaluated in this article, or claim that may be made by its manufacturer, is not guaranteed or endorsed by the publisher.
